# Socioeconomic status, severity of disease and level of family members’ care in adult surgical intensive care patients: the prospective ECSSTASI study

**DOI:** 10.1007/s00134-012-2463-x

**Published:** 2012-01-25

**Authors:** Thomas Bein, Kathrin Hackner, Tianya Zou, Sybille Schultes, Teresa Bösch, Hans Jürgen Schlitt, Bernhard M. Graf, Matthias Olden, Michael Leitzmann

**Affiliations:** 1 Department of Anaesthesiology and Critical Care, University Hospital, 93042 Regensburg, Germany; 2 Department of Epidemiology and Preventive Medicine, University Hospital, Regensburg, Germany; 3 Department of Surgery, University Hospital, Regensburg, Germany

**Keywords:** Socioeconomic status, Severity of disease, Adult surgical intensive care, SOFA score, SAPS II score, Outcome

## Abstract

**Background:**

Low socioeconomic status (SES) is associated with increased mortality from cardiovascular disease, cancer and trauma. However, individual-level prospective data on SES in relation to health outcomes among critically ill patients admitted to intensive care units (ICU) are unavailable.

**Methods:**

In a cohort of 1,006 patients at a 24-bed surgical ICU of an academic tertiary care facility in Germany, we examined levels of SES in relation to disease severity at admission, time period of mechanical ventilation, length of stay and frequency of phone calls and visits by next-of-kin.

**Findings:**

Patients with low SES had higher risk for Sequential Organ Failure Assessment (SOFA) score greater or equal to 5 [multivariate-adjusted odds ratio (OR) 1.49; 95% confidence interval (CI) 0.95–2.33; *p* = 0.029] and a trend for higher risk for Simplified Acute Physiology Score (SAPS II) greater or equal to 31 (OR 1.28; 95% CI 0.80–2.05; *p* = 0.086) at admission as compared with patients with high SES. When compared with men with high SES, those with low SES had greater risk for ICU treatment ≥5 days (multivariate-adjusted OR 1.99; 95% CI 1.06–3.74; *p* = 0.036) and showed a trend for a low number of visits from next-of-kin (<0.5 visits per day) (OR 1.85; 95% CI 0.79–4.30; *p* = 0.054). In women such associations could not be demonstrated.

**Interpretation:**

Socioeconomic status is inversely related to severity of disease at admission and to length of stay in ICU, and positively associated with the level of care by next-of-kin. Whether relations differ by gender requires further examination.

## Introduction

A graded inverse relation of socioeconomic status (SES) to disease and mortality has been demonstrated in a number of recent studies: at all levels of SES it was found that, the more advantaged individuals are, the lower their incidence of disease and mortality [1–4]. In a retrospective analysis involving over 400,000 trauma patients, race and insurance status emerged as predictors of mortality after trauma [5]. In cardiovascular patients, a strong inverse association between SES and incidence of vascular complications is apparent [6, 7]. SES is a categorisation based on education, occupation, income and availability of health-related and cultural resources [1], whereby higher SES confers resources needed to more effectively produce and maintain health over the life course [8].

In critical care patients, the relation of SES to health outcomes has been studied in several large retrospective cohorts; For example, Ho et al. [9] found that low SES was associated with increased risk of long-term mortality in Australia after adjusting for age, ethnicity and severity of illness. A positive association between social deprivation and hospital mortality among patients admitted to critical care units in England was reported by Welch et al. [10].

We investigated the relation of SES to severity of disease in a surgical intensive care unit (SICU). Specifically, we administered a questionnaire to patients or family members to assess the patients’ levels of education, occupation, income, marital and health insurance status, physical activity, smoking and alcohol use. In addition, physical and laboratory parameters were collected. The severity of disease was calculated using two well-established clinical scores. Furthermore, the frequency of phone calls or visits by next-of-kin was documented by the nursing staff throughout the intensive care treatment period. We hypothesized that low SES is associated with greater severity of disease at SICU admission, longer length of stay in the SICU, longer time period of mechanical ventilation and lower level of care by next-of-kin than high SES.

## Methods

The current study was approved by our local Institutional Review Board (Ethikkommission Universität Regensburg, no. 09/072). After obtaining written informed consent, an interview-based questionnaire was administered to next-of-kin after admission of patients to the SICU or directly to the patients after transferral to a normal ward. The questionnaire included information on education, occupation, income and social circumstances. In total, information regarding the following 12 variables was collected and recorded:

- Highest educational qualification
- Highest professional qualification
- Net monthly income (€)
- Nationality
- Number of persons per household
- Marital status
- Health insurance status
- Size of town of residence
- Smoking status
- Level of alcohol intake
- Usual physical activity level
- Medications prescribed prior to admission

### Assessment of socioeconomic status

SES was assessed using a multidimensional index [11, 12]. The index was calculated from patients’ next-of-kin responses regarding the following components: (1) patient’s education and professional qualification, (2) patient’s occupational position and (3) patient’s net household income. The education/professional qualification component included 17 response options, the occupational position component included 7 response options, and the net household income component included 9 response options. Each component was assigned a value of 1–7 points (low to high point value), yielding a total socioeconomic score of 3–21 points (low to high SES). Patients were grouped into three categories of SES: low SES (3–8 points of total socioeconomic score), intermediate SES (9–11 points) and high SES (12–21 points).

Additionally, the main diagnoses leading to intensive care admission were documented and the severity of disease was calculated using two well-established scores [Simplified Acute Physiology Score (SAPS II), Sequential Organ Failure Assessment (SOFA)]. SAPS II probability of mortality is based on patient variables recorded within the first 24 h of hospital stay (12 variables including a combination of physiological, laboratory and clinical variables) [13], while the SOFA scoring scheme assigns 1–4 points to each of the following organ systems depending on the level of dysfunction: circulatory, respiratory, renal, hepatic, haematology and central nervous system [14]. We used both the SOFA score and the SAPS II score because these scores each provide unique information regarding illness severity. Specifically, the SOFA score is based on fewer physiological parameters than the SAPS II score and it does not include information on the reason for admission or data on co-morbidity. By comparison, the SOFA score includes information on treatments such as vasopressors which is not assessed by the SAPS II score [15].

Furthermore, the length of stay at the SICU and the period of mechanical ventilation (“ventilator-free days within 28 days”) were documented. We do not report data on SES in relation to mortality because the number of fatal events in our study among patients for whom we collected sufficient SES data was small (*n* = 56 patients), which would have yielded imprecise risk estimates. Because the level of care by family members may positively affect the course of intensive care treatment and outcome [16], we performed comprehensive documentation of the frequency of phone contacts and visits by next-of-kin during the entire intensive care treatment of all patients for whom we obtained questionnaire information. Visits of groups of family members or visits by more than one person were counted as a single visit. Repeated visits with intermittent leaving of the hospital by next-of-kin were considered repeated visits and were enumerated according to their number. Our unit has flexible visiting hours, and the SICU nurse decided whether visits were appropriate based on the care situation and the patient’s condition. The frequency of visits was documented by the SICU nurse, the accuracy of which was assessed by frequent spot-checks by the study investigators. For statistical analysis, the absolute numbers of phone contacts and visits were divided by the number of days of intensive care treatment.

### Statistical analysis

After determination of low, intermediate or high SES, potentially confounding variables were classified according to the following groups: age (continuous); gender (men, women); marital status (single, married, separated/divorced, widowed); number of inhabitants of town residence (<1,000, 1,000–4,999, 5,000–9,999, 10,000–99,999, ≥100,000); health insurance status (statutory, private); smoking status (never, past, current); alcohol use (never, rarely, regularly); body mass index (<20.0, 20.0–24,9, 25.0–29.9, ≥30.0 kg/m^2^); physical activity (current, former, none); main diagnosis (surgery due to cardiovascular disease, surgery due to cancer, and non-cardiovascular/non-cancer diseases); and number of medications prescribed prior to admission (number of medications prescribed for cardiovascular disease, cancer and non-cardiovascular/non-cancer diseases, respectively). All such categorising was done before any modelling was conducted.

Multiple logistic regression was employed to calculate the odds ratios of severity of disease and level of care by family members within individual categories of SES using high SES as the reference group. The tests for linear trend were calculated by modelling the ordinal value of each category of SES as a single continuous variable. All odds ratios are presented with 95% confidence intervals, and all reported *p* values are two-tailed. The analyses were performed using SAS software release 9.2. High SOFA score was defined as SOFA score of 5 or greater according to Minne et al. [15], while high SAPS II score was considered as SAPS II score of 31 or greater [17].

## Results

A total of 1,197 interviews were performed consecutively with next-of-kin following admission of patients to our SICU between October 2009 and September 2010. We were unable to obtain sufficient information from 191 patients due to lack of informed consent (*n* = 96), early discharge from hospital (*n* = 24), lack of family members and delirium of the patient (*n* = 36) or other causes (*n* = 35). We considered the remaining 1,006 patients for further descriptive and analytic analyses.

The mean age in our patient cohort was 62 ± 16 years, and the predominant gender was male (64%). The main diagnoses leading to admission were cancer surgery (37.7%), surgery due to cardiovascular disease excluding heart disease (21.2%), trauma (11.7%), infection/sepsis (5.8%), acute respiratory insufficiency (3.8%), transplantation (3.4%), shock syndrome (3.2%), cerebral disorder (1.8%) and other diseases (11.4%). The mean SAPS (27.2 ± 11.1) and SOFA scores (4.0 ± 3.2) indicated moderate to critical disease severity within the first 24 h after admission. Admission due to emergency surgeries occurred in only 10% of the total patient group, and thus emergency patients were not analysed separately due to small numbers. The mean 28-ventilator-free-day score was 25.2 ± 5.3, with a mean 3 day period of mechanical ventilation. Mean duration of intensive care treatment was 5.9 ± 8.3 days.

Patients received 0.62 ± 0.43 phone contacts and 0.72 ± 0.61 personal visits by next-of-kin per SICU day.

**Fig. 1:**
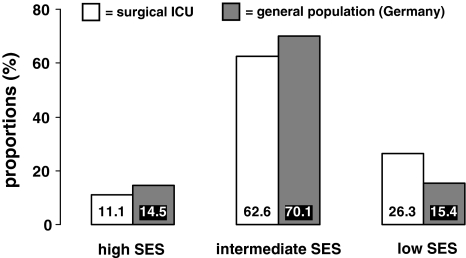
Proportions of patients with high, intermediate and low SES in comparison with the general population in Germany, 2009 [18]

Patient characteristics according to SES are presented in Table 1. The proportions of patients falling into the high, intermediate and low SES groups were 11.1%, 62.6% and 26.3%, respectively. This is consistent with a shift from the intermediate to the low SES status in our patient group as compared with the general population in Germany [18] (Fig. 1).

**Table 1:** Patient characteristics according to socioeconomic status

Characteristics	Socioeconomic status		
	High	Intermediate	Low
Patients (%)	11.1	62.6	26.3
Age (years)	57.8	61.1	61.9
Gender (%)			
Men	79.8	64.8	62.4
Women	20.2	35.2	37.6
Marital status (%)			
Single	11.7	13.7	22.9
Married	67.7	64.6	46.4
Separated/divorced	13.6	9.9	9.1
Widowed	7.0	11.6	21.5
Number of inhabitants of town of residence (%)			
<1,000	6.1	14.7	28.9
1,000–4,999	12.8	26.5	25.1
5,000–9,999	19.8	19.4	15.7
10,000–99,999	33.0	24.7	22.8
≥100,000	28.3	14.3	7.1
Health insurance status (%)			
Statutory	58.4	83.3	97.9
Private	41.1	15.4	0.3
Smoking (%)			
Never	31.9	32.1	41.5
Past	52.7	46.5	35.6
Current	15.3	21.2	22.9
Alcohol use (%)			
Never	16.0	26.8	37.7
Rarely	42.3	36.6	29.9
Regularly	41.7	36.6	31.9
Body mass index (kg/m^2^)	26.4	26.0	26.7
Physical activity (%)			
Yes, currently	38.3	28.3	13.4
Yes, formerly	25.7	29.0	18.7
No	22.0	30.4	53
Main diagnosis (%)			
Cardiovascular surgery (except heart disease)	30.7	40.6	39.5
Cancer surgery	43.0	35.8	38.1
Other	26.3	23.6	22.4
Number of medications prescribed prior to admission (*n*)			
Cardiovascular	0.2	0.1	0.1
Cancer	1.6	1.7	1.8
Other	0.5	0.5	0.5

All values (except age) were directly standardized to the age distribution of the patient group

As compared with patients in the high SES category, those in the low SES category were more likely to be older, to be of male gender, to be single or widowed, to be habitants of a small town of residence and to have statutory health insurance. Physical activity was inversely related to SES. No differences according to the SES distribution were noted with respect to the main diagnoses or to medications prescribed prior to admission. In contrast to findings from other studies [19], in our study patients with low SES were less likely to have smoked in the past and to consume alcohol than those with high SES.

In age-adjusted analysis, patients with low SES showed a statistically significant increase in risk for more severe disease status at admission as assessed by SOFA score (OR 1.50; 95% CI 1.01–2.22) as compared with patients with high SES (Table 2). Multivariate adjustment attenuated that risk estimate (multivariate OR 1.49; 95% CI 0.95–2.33), but the test for trend remained statistically significant (*p* for trend = 0.029). Likewise, low versus high SES was associated with increased SAPS II score by trend (multivariate OR 1.28; 95% 0.80–2.05; *p* for trend = 0.086) (Table 3). When we restricted our analyses to men, SES-related increasing risks for high SOFA score (multivariate OR 1.69; 95%, 0.96–3.00; *p* for trend = 0.048) and high SAPS II score (multivariate OR 1.90; 95% CI 1.02–3.55; *p* for trend = 0.061) emerged. No such relations were found in women.

**Table 2:** Age-adjusted and multivariate-adjusted odds ratio (95% CI) for SOFA score ≥5 in relation to socioeconomic status

Variable	Socioeconomic status			*P* for trend
	High	Intermediate	Low	
All patients				
No. of patients with SOFA score ≥5	31	234	107	
No. of patients with SOFA score <5	71	338	134	
Age-adjusted OR (95% CI)	1.0	1.26 (0.90–1.78)	1.50 (1.01–2.22)	0.013
Multivariate-adjusted OR (95% CI)	1.0	1.25 (0.87–1.82)	1.49 (0.95–2.33)	0.029
Men				
No. of patients with SOFA score ≥5	26	163	71	
No. of patients with SOFA score <5	52	210	76	
Age-adjusted OR (95% CI)	1.0	1.33 (0.87–2.02)	1.58 (0.97–2.58)	0.032
Multivariate-adjusted OR (95% CI)	1.0	1.35 (0.85–2.14)	1.69 (0.96–3.00)	0.048
Women				
No. of patients with SOFA score ≥5	5	71	36	
No. of patients with SOFA score <5	19	128	58	
Age-adjusted OR (95% CI)	1.0	1.17 (0.65-2.12)	1.37 (0.69–2.69)	0.192
Multivariate-adjusted OR (95% CI)	1.0	1.10 (0.56–2.16)	1.09 (0.49–2.43)	0.407

**Table 3:** Age-adjusted and multivariate-adjusted odds ratio (95% CI) for SAPS II score ≥31 in relation to socioeconomic status

Variable	Socioeconomic status			*P* for trend
	High	Intermediate	Low	
All patients				
No. of patients with SAPS II score ≥31	27	202	100	
No. of patients with SAPS II score <31	75	370	141	
Age-adjusted OR (95% CI)	1.0	1.08 (0.75–1.54)	1.36 (0.90–2.05)	0.027
Multivariate-adjusted OR (95% CI)	1.0	1.08 (0.73–1.59)	1.28 (0.80–2.05)	0.086
Men				
No. of patients with SAPS II score ≥31	21	136	62	
No. of patients with SAPS II score <31	57	237	85	
Age-adjusted OR (95% CI)	1.0	1.29 (0.82–2.04)	1.72 (1.02–2.91)	0.036
Multivariate-adjusted OR (95% CI)	1.0	1.40 (0.84–2.34)	1.90 (1.02–3.55)	0.061
Women				
No. of patients with SAPS II score ≥31	6	66	38	
No. of patients with SAPS II score <31	18	133	56	
Age-adjusted OR (95% CI)	1.0	0.77 (0.42–1.40)	0.90 (0.45–1.77)	0.353
Multivariate-adjusted OR (95% CI)	1.0	0.76 (0.39–1.47)	0.72 (0.33–1.59)	0.685

SES showed no statistically significant association (multivariate OR comparing low with high SES = 1.16; 95% CI 0.56–2.42; *p* for trend = 0.582) with duration of mechanical ventilation >6 days (equivalent to 28-day ventilator-free-days score <22). However, we found a statistically significant relationship between low SES and increasing length of stay in SICU (≥5 days) among men (multivariate odds ratio for low versus high SES = 1.99; 95% CI 1.06–3.74; *p* for trend = 0.036) (Table 4). Similarly, men with low SES showed a borderline statistically significant increasing risk for low number of visits from next-of-kin (multivariate OR 1.85; 95% CI 0.79–4.30; *p* for trend = 0.054) compared with men with high SES (Table 5). In women and in the all-patients cohort such an association could not be demonstrated. In addition, SES was unrelated to the number of phone calls received from next-of-kin during intensive care treatment.

**Table 4:** Age-adjusted and multivariate-adjusted odds ratio (95% CI) for length of stay ≥5 days in ICU in relation to socioeconomic status

Variable	Socioeconomic status			*P* for trend
	High	Intermediate	Low	
All patients				
No. of patients with stay in ICU ≥5 days	20	188	84	
No. of patients with stay in ICU <5 days	82	385	157	
Age-adjusted OR (95% CI)	1.0	1.60 (1.10–2.33)	1.79 (1.17–2.75)	0.011
Multivariate-adjusted OR (95% CI)	1.0	1.48 (0.98–2.24)	1.50 (0.91–2.46)	0.133
Men				
No. of patients with stay in ICU ≥5 days	16	132	56	
No. of patients with stay in ICU <5 days	62	241	91	
Age-adjusted OR (95% CI)	1.0	1.87 (1.17–2.99)	2.07 (1.21–3.52)	0.018
Multivariate-adjusted OR (95% CI)	1.0	1.83 (1.09-3.07)	1.99 (1.06–3.74)	0.036
Women				
No. of patients with stay in ICU ≥5 days	4	56	28	
No. of patients with stay in ICU <5 days	20	144	66	
Age-adjusted OR (95% CI)	1.0	1.10 (0.58–2.10)	1.21 (0.59–2.52)	0.387
Multivariate-adjusted OR (95% CI)	1.0	0.84 (0.40–1.80)	0.58 (0.24–1.43)	0.259

**Table 5:** Age-adjusted and multivariate-adjusted odds ratios (95% CI) for low number of visits per day (<0.5) and low number of telephone calls (<0.5) by next-of-kin in relation to socioeconomic status

Variable	Socioeconomic status			*P* for trend
	High	Intermediate	Low	
Visits per day by next-of-kin				
All patients				
Age-adjusted OR (95% CI)	1.0	1.25 (0.76–2.06)	1.72 (0.97–3.03)	0.017
Multivariate-adjusted OR (95% CI)	1.0	1.10 (0.64–1.90)	1.22 (0.64–2.33)	0.292
Men				
Age-adjusted OR (95% CI)	1.0	1.28 (0.68–2.40)	2.44 (1.19–5.01)	0.003
Multivariate-adjusted OR (95% CI)	1.0	1.22 (0.60–2.51)	1.85 (0.79–4.30)	0.054
Women				
Age-adjusted OR (95% CI)	1.0	1.32 (0.57–3.04)	0.88 (0.34–2.32)	0.752
Multivariate-adjusted OR (95% CI)	1.0	0.92 (0.35–2.42)	0.52 (0.17–1.66)	0.477
Telephone calls per day by next-of-kin				
All patients				
Age-adjusted OR (95% CI)	1.0	1.06 (0.68–1.65)	1.20 (0.71–2.02)	0.528
Multivariate-adjusted OR (95% CI)	1.0	1.13 (0.70–1.84)	1.28 (0.71–2.31)	0.565
Men				
Age-adjusted OR (95% CI)	1.0	1.15 (0.66–1.98)	1.57 (0.82–3.00)	0.120
Multivariate-adjusted OR (95% CI)	1.0	1.20 (0.64–2.22)	1.40 (0.66–2.98)	0.426
Women				
Age-adjusted OR (95% CI)	1.0	0.85 (0.39–1.85)	0.66 (0.26–1.68)	0.240
Multivariate-adjusted OR (95% CI)	1.0	0.96 (0.36–2.56)	0.64 (0.19–2.10)	0.250

## Discussion

We performed a prospective investigation of SES in relation to health outcomes using a 12-item questionnaire aimed at categorising SES based on an individual assessment of patients’ sociodemographic variables. To our knowledge, our investigation is the first prospective study of SES in relation to health outcomes in intensive care patients, and the main results are the following: (1) the proportion of patients with low SES was greater (26.3%) than that of the general population in Germany (15.4%) [18], consistent with a shift towards disadvantaged patients in the SICU, (2) low SES was found to be an independent predictor of high Sequential Organ Failure Assessment (SOFA) score at admission, with a similar trend seen for Simplified Acute Physiology Score (SAPS-II), and (3) on multivariate analysis, low SES was an independent predictor of long-term SICU treatment (≥5 days) in men, but no relation was observed for duration of mechanical ventilation (≥6 days).

In numerous studies [1–4], low socioeconomic status has been found to be associated with high incidence of diseases and worse health outcomes—even in developed countries. SES is defined as the sum of a number of sociodemographic variables such as gender, race, education, income and occupational status. Although a ‘unique’ pattern of SES has not been developed for all industrialised countries, low SES is related to increased all-cause mortality and specific causes of death, including cardiovascular disease and cancer [20, 21].

In three retrospective cohort studies [9, 10, 22], long-term mortality following intensive care was found to be significantly higher in patients from the lowest SES group in comparison with the highest SES group. While informative, those investigations were limited because their categorisation into SES groups was performed using aggregated data and no individual-level data regarding education, income or occupational status were assessed. By comparison, a recent retrospective cohort study [23] of 9,518 patients in adult ICUs of 35 California hospitals used individual-level data but found no differences regarding hospital mortality or ICU length of stay by race, ethnicity or SES, assessed by ZIP code.

The majority of patients in our study (64%) were male, and in other studies [8, 22] a similar gender proportion was found. In general, sex differences in morbidity and mortality have been known for a long time [24], such that remarkable discrepancies between health and survival exist between men and women. Men are physically stronger and have fewer disabilities, but they have substantially higher mortality at all ages compared with women, which has been referred to as the ‘male–female health–survival paradox’ [25]. In our study men represented nearly 2/3 of our patients, and this might partly explain our observation of a more pronounced relation of low SES to increased disease severity in men than in women.

We do not assume any impact of statutory versus private health insurance on the relation of SES to health outcomes in our study because, in Germany, chargeable health insurance fees for identical services do not differ for intensive care.

An additional aspect of our study was to assess the association between SES and the level of care (visits, phone calls) by next-of-kin. Available quantitative and qualitative data on family members’ care for adult patients are sparse: in one prospective investigation among 198 patients from a general ICU in Sweden, Eriksson et al. [26] found that 25% of patients had no visitors whatsoever, while 47% of patients with visitors had visits of ≤0.5 h/day, 36% had visits of between 0.6 and 2 h/day and 17% had visits of >2 h/day. We found that men with low SES were more likely to have a low number (<0.5/day) of visits compared with those with intermediate or high SES, while no significant SES differences were observed regarding the number of phone calls. Potential reasons for these findings include differences in marital status according to SES (Table 1) and perhaps other socioeconomic and psychological aspects that we were unable to assess (availability of day-care for children during visit of a parental family member to the SICU, available travel resources). The motivation for visiting a critically ill spouse or family member is influenced by emotional factors (uncertainty, emotional ‘roller coaster’, balance of hope and reality [27]) and financial resources. In a questionnaire administered to visitors of adult ICU patients in the UK, the mean cost of time forgone was 46 pounds sterling/visit and mean out-of-pocket expenses were 29 pounds [28].

Our study has some limitations. We did not examine mortality as an outcome because the number of fatal events in our study among patients for whom we initially were able to collect sufficient SES data was small. Secondly, we report data from a single study centre from a university hospital in Germany, which may limit the generalisability of our results.

In conclusion, we demonstrated that low SES is an independent predictor of severity of disease at admission to a surgical ICU. In addition, low SES was associated with prolonged length of stay in the ICU. Furthermore, the impact of low SES appeared to be more pronounced in men than women. A large multi-centre prospective study should confirm these results and include mortality as an outcome parameter.
